# Potassium Current Signature of Neuronal/Glial Progenitors in Amniotic Fluid Stem Cells

**DOI:** 10.3390/cells14010050

**Published:** 2025-01-04

**Authors:** Paola Sabbatini, Sabrina Cipriani, Andrea Biagini, Luana Sallicandro, Cataldo Arcuri, Rita Romani, Paolo Prontera, Alessandra Mirarchi, Rosaria Gentile, Diletta Del Bianco, Elko Gliozheni, Sandro Gerli, Irene Giardina, Maurizio Arduini, Alessandro Favilli, Antonio Malvasi, Andrea Tinelli, Bernard Fioretti

**Affiliations:** 1 Department of Chemistry, Biology and Biotechnologies, University of Perugia, Via dell’Elce di Sotto 8, 06123 Perugia, Italy; paola.sabbatini@unipg.it (P.S.); andrea.biagini@dottorandi.unipg.it (A.B.); luana.sallicandro@dottorandi.unipg.it (L.S.); rosaria.gentile@dottorandi.unipg.it (R.G.); dilettadelbianco@dottorandi.unipg.it (D.D.B.); elko.gliozheni@dottorandi.unipg.it (E.G.); 2 Rheumatology Unit, Department of Medicine, School of Medicine, University of Perugia, 06123 Perugia, Italy; sabrina.cipriani@unipg.it; 3 Department of Medicine and Surgery, University of Perugia, Piazza L. Severi 1, 06132 Perugia, Italy; cataldo.arcuri@unipg.it (C.A.); rita.romani@unipg.it (R.R.); paolo.prontera@ospedale.perugia.it (P.P.); alessandra.mirarchi@unipg.it (A.M.); sandro.gerli@unipg.it (S.G.); irenegiardina@hotmail.com (I.G.); maoard@libero.it (M.A.); alessandro.favilli@unipg.it (A.F.); 4 Laboratorio Interdipartimentale di Fisiopatologia della Riproduzione, Università degli Studi di Perugia, Edificio C, Piano 3 Piazza Lucio Severi, 1, Sant’Andrea delle Fratte, 06132 Perugia, Italy; 5 Department of Obstetrics and Gynecology, Faculty of Medicine, University of Tirana, AL1005 Tirana, Albania; 6 Centre of Perinatal and Reproductive Medicine, Department of Obstetrics and Gynecology, University of Perugia, 06123 Perugia, Italy; 7 Department of Biomedical Sciences and Human Oncology, University of Bari, 70121 Bari, Italy; antoniomalvasi@gmail.com; 8 Department of Obstetrics and Gynecology and CERICSAL (CEntro di RIcerca Clinico SALentino), Veris delli Ponti Hospital, Via Giuseppina delli Ponti, 73020 Scorrano, Italy

**Keywords:** amniotic fluid, neuronal/glial progenitors, neuronal differentiation, potassium channels, cell-based therapy

## Abstract

Amniotic fluid is a complex and dynamic biological matrix that surrounds the fetus during the pregnancy. From this fluid, is possible to isolate various cell types with particular interest directed towards stem cells (AF-SCs). These cells are highly appealing due to their numerous potential applications in the field of regenerative medicine for tissues and organs as well as for treating conditions such as traumatic or ischemic injuries to the nervous system, myocardial infarction, or cancer. AF-SCs, when subcultured in the presence of basic Fibroblast Growth Factor (bFGF), have been shown to survive and migrate when transplanted into the striatum of the rat brain, exhibiting behavior characteristics of neuronal/glial progenitor cells. In this work, we performed an electrophysiological characterization to ascertain the propensity of AF-SCs to differentiate into glial and neuronal cells by bFGF. By using patch clamp technique we characterized a fibroblast-like morphology that display a barium-sensitive inward-rectifying potassium current (Kir) and calcium-activated potassium currents (KCa). The electrophysiological and calcium dynamics of histamine, a marker of undifferentiated neural progenitors, was further studied. Histamine promoted intracellular calcium increase by Fura-2 recording and calcium-activated potassium current activation with a similar temporal profile in AF-SC. The data presented in this paper ultimately confirm the expression in AF-SCs of the Kir and KCa currents, also showing regulation by endogenous stimuli such as histamine for the latter.

## 1. Introduction

Human amniotic fluid, collected around the third month for prenatal diagnosis of congenital anomalies, contains cells of fetal origin, which can be easily made adherent and cultured for a few passages [1,2]. Numerous studies indicate that these cells can be divided into three major groups based on their morphological, biochemical and growth characteristics, the relative proportion of which changes with the gestation period [3,4]: epithelioid cells (type E), amniotic fluid cells (type AF) and fibroblastic cells (type F). Amniotic fluid is a source of pluripotent stem cells capable of giving rise to multiple cell types representative of all three embryonic germ layers [5,6]. Amniotic fluid stem cells (AF-SCs) have rapidly attracted attention for their potential to differentiate in vitro into several diverse types of cells, including cardiac, osteogenic, skeletal muscle, bone, cartilage, lung, ovarian, hepatic and neuronal cells, resulting particularly promisingly in the field of regenerative medicine for in vivo reconstruction of bio-artificial tissues and organs [7]. Scar-free wound healing is another investigated application of AF-SC, for their anti-inflammatory and immunomodulatory ability that protect the wound microenvironment [8]. Other promising results have been obtained in the field of treatment of myocardial infarction [9], ischemic injuries of the central nervous system [10] and cancer [11].

Regarding their neurogenic properties, AF-SCs have shown interesting neurogenic potential; that is, the ability to differentiate into all cells of the nervous system. In vitro studies have shown that AF-SCs can be induced to differentiate into neuronal and glial cells [12,13] using specific growth factors and molecular signals that mimic the nervous system environment. Furthermore, AF-SCs can secrete neurotrophic factors, molecules that support the growth, survival and differentiation of neurons. These factors can have beneficial effects in the microenvironment of damaged nervous tissue, promoting repair and regeneration [14]. These cells also have the potential to help regenerate damaged nervous tissue. In animal models, AF-SCs transplanted into areas of neuronal injury have shown the ability to integrate into host tissue, promoting regeneration and improving neurological function [15,16]. Recently, human MSCs obtained from different sources have been employed in clinical trials for the treatment of various nervous system disorders, including ischemic stroke, spinal cord injuries, but also Alzheimer disease, amyotrophic lateral sclerosis and multiple sclerosis. These trials have yielded promising initial results, with improvement of the clinical conditions and no discernible adverse effects on short term [17]. However, it must be acknowledged that this technique is still constrained by numerous limitations, both technical and related to the route of administration. Technical limitations include the difficulty of obtaining sufficient quantities of cells, while the potential for cells to differentiate into tumor-like cells during the proliferative phase represents a further challenge [17].

It is known that there is a population of neural stem cells (NSCs) that express glial markers, such as the glial fibrillary acidic protein (GFAP) [18]. Neural stem cells are multipotent progenitor cells that are present in specific areas in the adult brain such as the subventricular zone of the lateral ventricles and the subgranular zone of the dentate gyrus of the hippocampus [19]. In these regions, a subpopulation of neural stem cells was observed to express GFAP, which is traditionally considered a marker of astrocytes [20]. The expression of glial markers in neural stem cells therefore suggests that these cells have astrocytic characteristics. AF-SCs show common traits with NSCs/neural progenitor cells, but further molecular characterization is needed to define the differentiation status of AF-SCs.

Potassium (K^+^) channels, through their control of the membrane potential by controlling Ca^2+^ influx and cell volume, play a very important role in cell proliferation and differentiation [21]. During the last decade, several studies have been performed in order to achieve the electrophysiological characterization of AF-SCs [22]. In 2016, Iordache et al. performed electrophysiological recordings of two populations of AF-SCs, cryopreserved or cultured for 6 weeks, obtaining a senescence model of the cells [23]. Interestingly, they reported that a subset of cryopreserved cells expressed a typical large inward rectifier K^+^ current that was completely lost in senescent cells. These cells, which account for 30% of cryopreserved cells, were also featured by neuronal/glial progenitor marker expression (tubulin b-III, NF-200 and CD56/NCAM found by flow cytofluorimetry), suggesting a central role of ionic currents in driving neuronal differentiation. However, further studies are needed to understand the possible role of K^+^ currents in the proliferation and differentiation of AF-SCs.

In this work we present the electrophysiological characterization of a population of fibroblastic-like AF-SCs collected at 3 months during an amniocentesis procedure and cultured in the presence of bFGF [24]. This population of AF-SCs were selected because previous studies have shown that, when transplanted into the corpus striatus of normal and ischemic rats, they were able to survive, migrate and differentiate in astrocytic cells [24]. These cells were reported to express the astrocytic markers GFAP at 10, 30 and 90 days after transplantation, suggesting their ability to differentiate and integrate into the host [24]. These promising results prompted us to investigate what the biological basis of this behavior is by electrophysiological evaluation.

## 2. Materials and Methods

### 2.1. Isolation and Culture of AF-SCs

Human amniotic fluid stem cells were obtained from human amniotic fluids of pregnant women (aged 35–40 years) in their 16th or 17th week, who underwent amniocentesis during routine prenatal diagnosis. The study was approved by the University of Perugia Bioethics Committee, and each participant provided informed consent for the secondary use of amniotic fluid samples. The isolation was performed according to Romani et al. [25]. Briefly, residual cells from prenatal diagnosis tests were cultured in mesenchymal stem cell growth medium (MSCGM, Lonza Gaithersburg, Rockville, MD, USA) for one week. The isolation of AF-SCs consisted in selecting the cultures containing cells with peculiar morphology and colony shape. These colonies were then selected and cultured for several passages in vitro [25]. After isolation and characterization, the cells were plated at 1000 cells/cm^2^ in Iscove’s modified Dulbecco’s medium (IMDM) (Invitrogen, Carlsbad, CA, USA) supplemented with 10% FBS, 10 ng/mL bFGF and 2 mM L-glutamine, according to Cipriani [24,26]. When the cell cultures were about 50–60% confluent, they were detached and re-plated at 1:3 under the same culture conditions and cultured with two weekly medium changes.

### 2.2. Electrophysiological Recordings

Ion currents in human AF-SCs were recorded by using the patch clamp technique in a whole-cell dialyzed patch clamp configuration. Currents were amplified with a HEKA EPC-10 amplifier and analyzed with the PatchMaster (www.heka.com) and Origin 4.1 software. For online data collection, currents were filtered at 3 kHz and sampled at 40 μs/point. The modified Ringer external solution contained (mM) NaCl 106.5, sodium gluconate 15, KCl 5, CaCl_2_ 2, MgCl_2_ 2, MOPS 5 and glucose 20, at pH 7.25. The pipette solution contained (mM) K-aspartate 114, MgCl_2_ 2, CaCl_2_ 0.08, MOPS 5 and EGTA-K 1, at pH 7.25. Electrical access ranged between 10 and 20 MΩ after cell membrane breaking. In the perforated-patch configuration, electrical access to the cytoplasm was achieved by adding amphotericin B (200 μM) to the pipette solution. Access resistances ranging between 15 and 25 MΩ were achieved within 10 min following seal formation and were actively compensated to ca. 50%. Amphotericin B was dissolved in DMSO to a concentration of 50 mM.

### 2.3. Indirect Immunofluorescence

Cells were extensively washed with phosphate-buffered saline (PBS), immersed in cold methanol, kept at −20 °C for 7 min, permeabilized wih 0.1% Triton X-100 in PBS for 10 min and washed three times in PBS. After overnight incubation with blocking buffer (3% bovine serum albumin, 1% glicine in PBS), the cells were incubated for 60 min at room temperature with a rabbit anti-GFAP (Dakopatts, Glostrup, Denmark) polyclonal antibody (diluted 1:100 in PBS containing 3% bovine serum albumin) and washed three times in PBS 0.1% Tween 20 and two times in PBS. After treatment with tetramethylrhodamine isothiocyanate-conjugated (Sigma-Aldrich, St. Louis, MO, USA) goat anti-rabbit IgG (diluted 1:100 in PBS containing 3% albumin), three washes with PBS containing 0.1% Tween 20 and two washes with PBS, the preparations were incubated with 2 μg/mL DAPI (4,6-diamidino-2-phenylindole; Sigma) for 1 min and dried in air. Coverslips were mounted and the preparations observed with a DMRB Leika microscope equipped with a digital camera.

### 2.4. Cytosolic Ca^2+^ Measurements

According to our previous work [27], cells were incubated with FURA-2-AM (3 μM; Sigma-Aldrich, St. Louis, MO, USA) for 45 min and extensively washed with external Ringer’s solution of the following composition: 140 mM NaCl, 2.5 mM KCl, 2 mM CaCl_2_, 2 mM MgCl_2_, 5 mM MOPS and 10 mM glucose, at pH 7.4. Cells were continuously perfused using a gravity-driven perfusion system, focally oriented onto the field of interest. The estimation of intracellular free Ca^2+^ concentration was reported as the change in the ratio between fluorescence emission at 510 nm obtained with 340 and 380 nm excitation wavelengths (optical filters and the dichroic beam splitter were from Lambda DG4, Shutter Instruments). Ratiometric data were acquired every 3 seconds, and fluorescence determinations were performed using fluorescence microscopy system Zeiss (Axiozoom V16 and Axiocam 502 mono). The intracellular calcium variation after histamine perfusion (100 μM) was estimated by calculation of the 340/380 fluorescence ratio of FURA-2.

### 2.5. rt-PCR

RNA was extracted using TRIzol™ (Invitrogen), according to the manufacturer’s instructions, followed by cDNA synthesis by reverse transcription using the QuantiTect^®^ Reverse Transcription Kit (Qiagen, Shenzhen, China). The quality and quantity of RNA were evaluated on the Infinite M Nano+ spectrophotometer (Tecan, Männedorf, Switzerland). cDNA analysis was performed through rt-PCR with the SYBR^®^ Green PCR Kit (Qiagen) according to the manufacturer’s instructions, operated by the Rotor-Gene Q machine (Qiagen). The sequences of the primers used are reported in Table 1. The reference gene Actin Beta was used for housekeeping. The reaction conditions were as follows: 2 min at 95 °C for an initial denaturation and enzyme activation, followed by 35 cycles with denaturation at 95 °C for 5 s and annealing/elongation at 60 °C for 10 s. Negative controls (NTCs) were treated with RNA-free water in place of cDNA. All samples were amplified in triplicate. Primary specificity was assessed by the melting curve analysis. Gene expression levels were normalized to the reference gene, by using the threshold method.

### 2.6. Statistical Analysis

All data are reported as the mean ± standard error (SE). The Student’s t test was used to evaluate differences, and *p* < 0.05 was considered statistically significant. Statistical analyses were performed with Origin 6.1 software (OriginLab Corporation, Northampton, MA, USA).

## 3. Results

### 3.1. Functional Expression of Kir Currents and Their Pharmacological Profile

**Figure 1:**
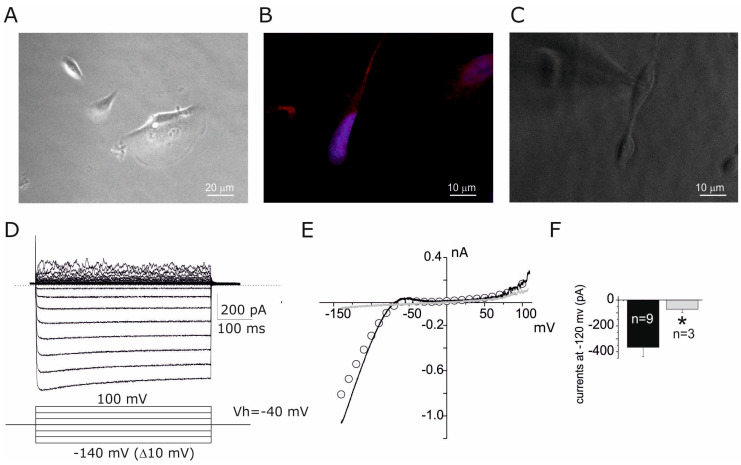
Expression of Kir currents in amniotic fluid cells with a fibroblastic morphology. (**A**) Representative microscopic field displaying the heterogeneous morphology of our cellular preparation; (**B**) GFAP immunofluorescence staining of a fibroblastic-type morphology observed in our cell preparation, with blue fluorescence due to DAPI nuclear staining; (**C**) a cell with fibroblast-like morphology during electrophysiological recording, note the microelectrode. (**D**) A family of currents recorded from a Vh of −40 mV with depolarizing pulses from −140 mV to +100 (with 10 mV increments) at 500 ms. Note the large inward component compared to the outward component, and the noise of the current traces at positive potentials. (**E**) The I-V relationship of the traces shown in (**D**) (dot points) constructed by plotting the peak currents as a function of the pulse test. The black solid line represents the current obtained by applying a potential ramp from −140 to +110 (Vh −40 mV) in the same cell as (**D**). The gray trace is the I-V relationship recorded from a cell with insignificant Kir current by using the ramp protocol described in (**E**). (**F**) Bar plot of currents at −120 mV derived from cells with (*n* = 9) and without Kir (*n* = 3). * *p* value < 0.05.

Cells derived from amniotic fluid show an evident morphological heterogeneity (Figure 1A), with a predominance of fibroblastic-type cells, and a minority of cells with other morphologies, such as small cubic cells (probably of an epithelial nature) and cells with a migratory morphology (with evident lamellipodium) [28,29]. In this work, we focused on fibroblastic type cells that, under our experimental conditions, express Glial Fibrillar Acidic Protein (GFAP, Figure 1B) according to an in vivo transplantation study [24]. Electrophysiological recordings using the patch clamp technique (Figure 1C) in a whole-cell dialyzed configuration were performed by applying, in voltage-clamp mode, potential pulses of a 500 ms duration from −140 mV to +100 mV (V holding −40 mV, down inset Figure 1D). A typical family of currents recorded with this protocol from a cell derived from amniotic fluid is shown in Figure 1D. A large inward current is evident for membrane potentials below −70 mV typically associated with inward potassium currents (Kir, see below). The current–voltage relationship (I-V) for the current family shown in Figure 1D was constructed by plotting current as a function of applied potential (dot points Figure 1E) superimposed with the currents obtained with the linear voltage gradient (ramp protocol) of potential, exploring a similar voltage range (Figure 1E, black line). The I-V relationship obtained by this method is similar to that obtained through discrete pulse analysis; therefore, this protocol was selected for the following investigations. Kir currents were observed in 75% (9/12) of the cells with a fibroblast morphology, whereas the other cells do not express Kir currents (Figure 1E, gray trace). Comparison of Kir currents between the two groups of cells was performed at −120 mV (Figure 1F), resulting in a significative difference between the two subpopulations (*p* value < 0.05). At positive potentials (>50 mV), a delayed rectifier type of voltage-gated outward potassium (DRK) current (with an activation threshold >+50 mV) with marked noise was observed (see below).

**Figure 2:**
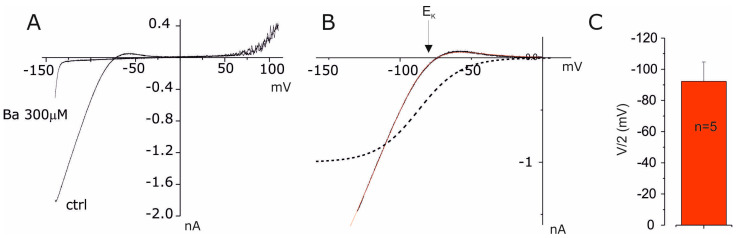
Activation properties of Kir currents in fibroblastic AF-SCs. (**A**) The current–voltage relationship obtained by the ramp potential protocol (−140 to +110, Vh −40 mV) under control conditions and in the presence of Ba^2+^ (300 µM). (**B**) The I-V relationship of the Ba-sensitive current obtained by subtraction of the control traces minus the one in Ba^2+^ shown in (**A**). The solid red line represents the best fit with the Boltzmann equation: I = (G*(V − E_k_))/1 + e^(V−V/2)/k^, where G is the macroscopic conductance, E_k_ is the equilibrium potential of potassium, V/2 is the gating charge and half-activation potential and k is the voltage sensitivity. The dotted line represents the normalized Boltzmann function obtained from the fit (red line). (**C**) The bar plot of mean V/2 obtained from five experiments similar to that described in (**B**).

In order to further characterize the Kir currents, we studied the barium (Ba^2+^) ion blocking activity [30]. The 300 µM Ba^2+^ fully blocked inward currents, while no effects were observed on the outward component (Figure 2A); accordingly, with voltage dependences of the barium block that increase at negative voltages, where Kir currents are operative, and nullified at positive voltage where DRK currents act [31]. As can be seen from the difference in the ramp currents, expanded in the potential range from −130 to 0 mV, the reversal potential of barium-sensitive currents is a value very close to the equilibrium potential of K^+^ ions under the recording conditions used (E_K_ = −89 mV), suggesting that this current is supported by K^+^ channels (Figure 2B). In order to study the voltage dependence of the activation of this current, we analyzed the current using a function that takes into account the Boltzmann relation and the electromotive force (fem) in order to obtain the half-activation potential and the k constant. The results of this analysis indicate that the inward current in amniotic fluid cells is activated in the direction of hyperpolarisation with a V/2 of −87 mV and a k of 14.8 (Figure 2B). The results from other similar experiments yielded a profile comparable to that shown in Figure 2B, with a mean V/2 activation of −92 ± 12 mV and a k value of 19 ± 6 (Figure 2C, *n* = 5).

**Figure 3:**
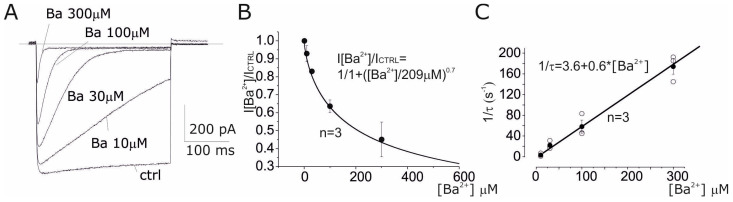
The dose and voltage dependences of the Kir Ba^2+^ block in amniotic fluid cells. (**A**) Inward currents recorded by applying a hyperpolarising pulse at −130 mV from a Vh of −40 mV with a duration of 500 ms, under control conditions (ctrl) and various concentrations of Ba^2+^ (10, 30, 100 and 300 µM). Note the presence of an instantaneous block (peak) and of a second block that develops during the hyperpolarization test. (**B**) Dose dependence of mean instantaneous peak currents (*n* = 3) obtained from similar experiments to those shown in (**A**). The black line represents the better fit with Hill’s equation. (**C**) The relationship between the mean reciprocal of the inactivation constant (*n* = 3) at various Ba^2+^ concentrations obtained from experiments similar to those shown in (**A**). The black line represents the better linear fit (see text for details).

The Ba^2+^ effect was further investigated using different concentrations, evoking Kir currents by a hyperpolarising pulse of −130 mV (500 ms). Under control conditions, the Kir current showed rapid activation (<5 ms) and relatively slow, partial inactivation (Figure 3A, ctrl). Ba^2+^ induced a blockade of the Kir currents in a time- and concentration-dependent manner. More specifically, it was observed that, in the presence of Ba^2+^, the Kir current could still be activated by the hyperpolarising pulse, while it was inhibited during the test pulse. This result suggests a voltage-dependent blocking mechanism where Ba^2+^ increases the potency of the block with hyperpolarization. We estimated the voltage-dependent block by evaluating the dose–response at two voltages: −40 mV (assessing the instantaneous current evocated during the hyperpolarizing pulse) and −130 mV (analyzing the kinetics of the block during the hyperpolarizing pulse). At −40 mV, the potency of the block was dose-dependent with a Kd of 209 μM (Figure 3A,B). The blocking mechanism at −130 mV was estimated from the inactivation process seen in the presence of Ba^2+^, as was the process of the binding of the ion to the open channels, according to the kinetic scheme:

C + Ba ↔ CBa,(1)

where C is the channel and CBa the channel–Ba complex. Equation (1) predicts that the inactivation phenomenon seen in the presence of Ba^2+^ follows an exponential trend of the type

I = A exp(−t/τ) (2)

where τ is the blocking time constant. The τ constant and the kinetic constants, k and l, are already described by the relationship

1/τ = k [Ba] + l(3)

where k and l are the kinetic constants of the attack and detachment of the Ba^2+^ ion from the channel [31]. In order to derive the values of k and l, the inactivation of the K^+^ current at various Ba^2+^ concentrations was analyzed with a single exponential function, and the inverses of the derived time constants were graphed as a function of Ba^2+^ concentration obtained from three similar experiments (Figure 3C). As predicted by the proposed blocking mechanism, the mean value obtained was well described by a linear regression with an angular coefficient (k) of 0.6 and an intercept (l) of 3.6. Finally, knowing the values of k and l and using the relationship Kd = l/k, we calculated the mean dissociation constant for blocking by Ba^2+^ as 6 µM.

### 3.2. Functional Expression of KCa Currents and Their Regulation by Histamine

**Figure 4:**
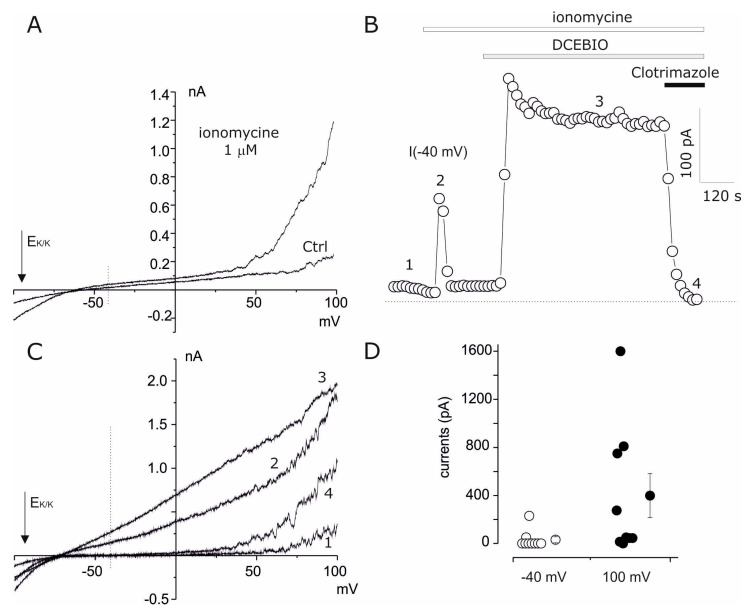
Calcium-activated potassium currents in amniotic fluid cells. (**A**) Current ramps obtained by applying linear potential gradients from −140 to 110 from a Vh of −40 mV recorded in ctrl and after ionomycin 1 μM application. Note the increase in outward currents caused by the shift of threshold in voltage activation at negative potentials and characterized by noise. (**B**) The time course of the −40 mV current measured from current ramps as described in (**A**) repeated every 15 s. The timing of ionomycin, DCEBIO + ionomycin (100 µM + 500 nM) treatment and co-application with clotrimazole (2 µM) are shown with the bar in the upper part of the graph. Note the transient activation (run-down) of currents during ionomycin application. (**C**) Current ramps recorded at the times indicated in (**B**) under different conditions: CTRL (1), ionomycin (2), DCEBIO+ionomycin (3) and DCEBIO + ionomycin+ clotrimazole (4). The arrows in (**A**,**C**) indicate the reversal potential of potassium currents in our condition whereas the vertical dashed line indicates −40 mV. (**D**) A scatter plot of the current activates by DCEBIO + ionomycin at −40 mV (empty dots) and +100 mV (black dots).

Previous studies characterized the expression of big conductance calcium-activated potassium (BK_Ca_ Iuphar name Kca1.1) currents in amniotic cells [23,32] and we investigated whether outward currents observed at a membrane potential > 50 mV (Figure 1) could represent these current subfamilies [23]. In order to study the calcium-activated potassium currents in amniotic fluid cells, we investigated the electrophysiological effects of intracellular calcium elevation promoted by ionomycin. Ionomycin 1 μM decreased the threshold of activation of the voltage-dependent outward currents at negative potentials, according to the voltage and calcium activation of BK_Ca_ currents (Figure 4A) [33]. We also observed a transient current activation at −40 mV, typically associated with the run-down process detected after ionomycin’s activation of intermediate conductance calcium-activated potassium (IK_Ca_, Iuphar name KCa3.1) currents [34] (Figure 4B,C). As expected, a stable IK_Ca_ current activation was observed by co-application of the activator of KCa (IK_Ca_/SK_Ca_) currents, 5,6-dichloro-1-ethyl-1,3-dihydro-2H-benzimidazol-2-one (DCEBIO) [35] with ionomycin (Figure 4B). In line with the expression of IK_Ca_ currents, the selective IK_Ca_ current blocker clotrimazole [36] fully blocked the DCEBIO/iono-activated currents at −40 mV (Figure 4B,C), while leaving the currents at +100 mV associated meanly with BK_Ca_ that was fully blocked by 3 mM tetraethylammonium (TEA). The IK_Ca_ and BK_Ca_ currents were estimated in nine cells after activation by DCBEIO + ionomycin coapplication, at −40 mV associated mainly to IK_Ca_, and at +100 mV associated to both currents (IK_Ca_ and BK_Ca_). In total, 2/9 of the studied cells clearly displayed IK_Ca_ currents, whereas BK_Ca_ was clearly identify in virtually all cells, with a high expression in half of the cells tested (4/9) (Figure 1D). BK_Ca_ expression was also confirmed from a molecular point of view by rt-PCR quantification (threshold values: ACTB 16.46 ± 0.029, *n* = 3; KCNMA1BK ALPHA 26.5 ± 0.96, *n* = 3). Notably, Kir current is inhibited by the coapplication of DCEBIO+ionomycin, but this effect was not further investigated.

**Figure 5:**
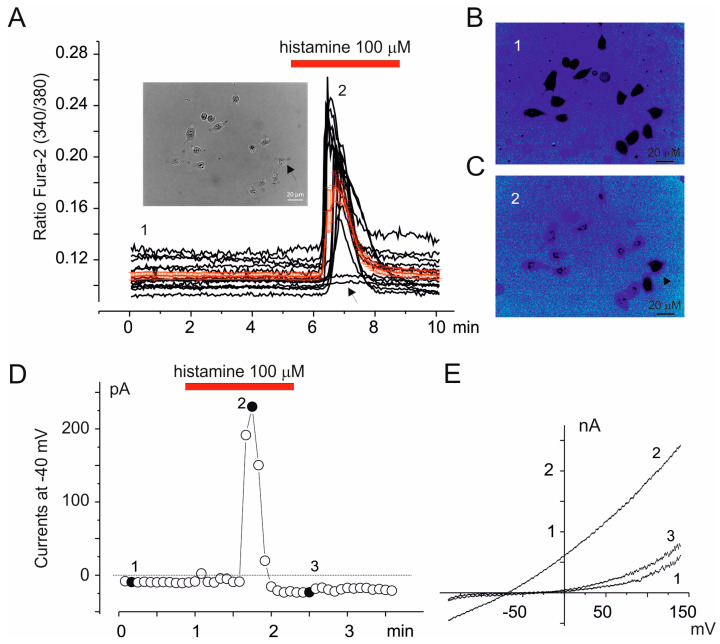
Histamine increases intracellular calcium and activates KCa. (**A**) The exemplificative calcium imaging experiment that displays the effects of the application of 100 mM of histamine in amniotic cells evaluated by Fura-2-based calcium imaging. The single black traces represent the single-cell recording of the Fura-2 signal recorded in each ROI (region of interest) as indicated with circles in the inset. The red line represents the mean of all traces (*n* = 14). (**B**,**C**) Representative frames of Fura-2 fluorescence recorded at the time indicated in panel (**A**) before (1) and at the peak of the response to histamine (2), respectively. Note the presence of unresponsive cells (arrows in **A** and **C**). (**D**) The effects of the application of 100 μM of histamine on outward currents at −40 mV during whole-cell perforated configuration recording. (**E**) The I-V relationships obtained by applying voltage ramp protocols from −140 to 140 (Vh = −40 mV) recorded before (1), at the peak (2) and after peak (3) of histamine activation, respectively, as reported in (**D**).

An undifferentiated neural progenitor application of 100 μM histamine increases intracellular calcium concentration through histamine 1 (H1) receptors [37,38]. Similarly, the application of histamine (100 μM) induced a generally transient intracellular calcium increase, evaluated by using Fura-2-based calcium imaging, in almost all of the cells analyzed (Figure 5A–C). Using the whole-cell perforated configuration to maintain the integrity of cytoplasmic cell signaling components [33], we tested the electrophysiological effects of histamine application in AF-SCs. According to calcium imaging experiments, histamine transiently increased calcium-activated potassium (K_Ca_) currents associated with IK_Ca_ and BK_Ca_ currents (Figure 5D,E).

## 4. Discussion

Among the morphologically heterogeneous cells derived from amniotic fluid, we focused on a fibroblast-like morphology that probably represents F-type cells, whose origin has been assumed to be from the connective tissue and dermal fibroblasts of the fetus [3]. The neurogenic potential of these cells makes them interesting candidates for the development of therapies for neurodegenerative diseases such as Parkinson’s disease, amyotrophic lateral sclerosis (ALS) and spinal cord injuries [39]. In fact, several preclinical studies have explored the use of AF-SCs in models of neurological diseases. For example, in animal models of ischemic stroke, transplanted AF-SCs showed improvements in brain tissue regeneration and the recovery of motor functions [40]. Moreover, a recent study explored the therapeutic potential of exosomes derived from human AF-SCs for protecting microglial and neuronal cells from the toxic effects of the amyloid-beta (Aβ) peptide, which is associated with Alzheimer’s disease [41]. AF-SC-derived exosomes significantly reduce Aβ-induced toxicity in neuronal and microglial cells and the viability of exosome-treated cells is higher than untreated cells. Exosomes exhibit an anti-inflammatory effect, reducing the production of pro-inflammatory cytokines by microglial cells. The molecular mechanisms involved in neuroprotection include the modulation of cell signaling pathways that promote cell survival and reduce oxidative stress.

Considering the huge potentiality of AF-SCs in the treatment of nervous system pathologies, in the present study we investigated the electrophysiological characteristics of these cells when they differentiate into the neuronal lineage. In particular, we used F-type AF-SCs cultured in the presence of bFGF that were able to survive, migrate and differentiate in astrocytes when transplanted in the corpus striatus of normal and ischaemic rats [24]. From an electrophysiological point of view, this sub-population is fairly homogeneous. The study of the current–voltage relationship showed that these cells are typically characterized by the expression of voltage- and calcium-activated potassium currents (K_Ca_) and potassium rectifying inward currents (Kir). Among K_Ca_ currents, the pharmacological and biophysical properties found were consistent with the expression of Big (BK_Ca_) and Intermediate (IK_Ca_) conductance calcium-activated potassium currents. In line with this conclusion, currents recorded at positive potentials are characterized by high noise, which is typical of channels with a high conductance of BK_Ca_. Co-application of DCEBIO, the activator of these currents, and ionomycin activates in the same cells a voltage-independent potassium current that is clotrimazole-sensitive, typically associated with IK_Ca_. Previous studies have already reported different ion current components, in particular BK_Ca_, IK_Ca_ and Kir currents, in AF-SCs submitted to cryopreservation protocols or during their differentiation toward cardiomyocyte cells [22,23]. More interestingly, a fast voltage-dependent Na+ current was reportedly co-expressed with Kir current in cryopreserved AF-SCs but further study is need to confirm the sodium currents in our experimental condition [23].

The co-expression of calcium-activated potassium channels of intermediate (IK_Ca_) and large conductance (BK_Ca_), is a feature found in many other preparations, such as macrophages, endothelial cells, some glandular cells and glioblastoma tumor cells [33,42]. Recently, we also proposed a link between the increase in IK_Ca_- and BK_Ca_-activated calcium currents and gliomagenesis [43]. The presence of IK_Ca_ channel currents in bFGF-cultured AF-SCs is of considerable interest, given their role in many proliferative and differentiative processes. There would thus seem to be a strong electrophysiological analogy between the fibroblastic subpopulation of amniotic cells and the glioblastoma cell line. Since the cells analyzed show a high percentage of immunopositivity for GFAP and simultaneously for neuronal markers such as β-III tubulin and the presence of stem markers such as CD133, as well as complete immunopositivity to nestin [24], we advance the hypothesis that these cells are embryonic neuronal precursors. To confirm this possible stemness, stem cell transplants in infarcted brains have shown the migration of cells with an astrocytic phenotype of human amniotic derivation [3,24].

With particular reference to glioblastoma cells, we note that a subpopulation of them also expresses Kir current-associated Kir4.2. A characteristic feature of AF-SCs is the presence of a strongly inward-rectifying (Kir) current. Although seven gene subfamilies capable of generating Kir currents in expression systems have been cloned, the Kir 4.2 subfamily is characterized by a voltage-dependent block by barium with a potency similar to that reported in this work [44]. We therefore tentatively attribute the current present in amniotic fluid cells to this subfamily. With reference to Kir 2.x, four members have been cloned: Kir 2.1, 2.2, 2.3 and 2.4, which can co-assemble as homotetramers or as heterotetramers [45]. The sensitivity to Ba^2+^ can help to understand the molecular basis and stoichiometry of the Kir currents expressed in AF-SCs. Indeed, given the high sensitivity to Ba^2+^ (Kd = 6 µM), we can rule out the possibility that this current is formed by homotetrameric channels or the Kir 2.3 or Kir 2.4 subunits. In fact, these channels are hardly susceptible to Ba^2+^ (IC_50_ of about 10 and 50 µM, respectively). In addition, the homotetrameric channels for the Kir 2.4 subunit are time-independently blocked by Ba^2+^, whereas the blocking of our currents exhibits a characteristic time dependence [30]. Although we cannot reach the exact molecular basis and stoichiometry of our Kir currents, we can speculate that the Kir4.1, Kir4.2, Kir 2.1 and Kir 2.2 subunits must be present as these confer a Ba^2+^ sensitivity similar to ours and time-dependent blocking. With regard to blockade by Ba^2+^, our current differs from that reported by De Coppi et al. [5] in a subpopulation of amniotic cells, which was attributed to Kir 3.2 (GIRK) channels. In fact, this current is partially sensitive to Ba^2+^ and the blocking mechanism appears to be time-independent. Our currents can hardly represent GIRK currents as we observe these currents in the absence of any agonist and their basal activity in the absence of G protein is negligible, as already reported by other groups [46]. A similar consideration can be made for Kir 6 channels and the molecular basis of K_ATP_ channels. Although Kir currents, known for setting the resting potential in the vicinity of the potassium equilibrium potential, are expressed in amniotic fluid cells, the resting potential of these cells, estimated by means of the current clamp mode, is relatively depolarized (approx. −20 mV). One possible explanation could be that these cells also co-express high amounts of a cationic current along with the Kir currents, which would counteract the hyperpolarizing effect of the Kir currents. Further studies will be necessary to conclusively address the molecular nature of Kir currents expressed in neuronal/glial progenitors in AF-SCs.

In astrocytes, Kir (inward rectifier potassium) and calcium-activated potassium (KCa) channels play crucial roles in maintaining potassium homeostasis and regulating calcium signaling, both of which are vital for proper brain function. Astrocytes are non-excitable glial cells, but their ability to buffer potassium ions and regulate calcium-dependent processes is essential for maintaining the extracellular environment necessary for neuronal activity [47,48]. Kir channels in astrocytes, particularly Kir4.1, are the primary mechanism by which astrocytes buffer excess potassium from the extracellular space, especially during neuronal activity. This potassium buffering helps regulate the resting membrane potential and protects neurons from hyperexcitability, which is crucial in conditions such as epilepsy [49,50]. Kir4.1 is concentrated around synapses and blood vessels, allowing astrocytes to spatially buffer potassium and maintain proper brain ion homeostasis [51]. The interaction between Kir and K_Ca_ channels in astrocytes is critical for controlling calcium waves, which are a form of astrocytic signaling that influences neuronal function and blood flow in the brain [48,52]. Kir channels maintain membrane potential, which influences calcium influx and, in turn, activates K_Ca_ channels. This dynamic interaction allows astrocytes to respond to neuronal activity and participate in processes such as neurovascular coupling and the modulation of synaptic transmission [51,53].

Our results, in conclusion, provide insight into the types of potassium currents that occur in AF-SCs that are predisposed to the development of neuronal lineage, which can be used therapeutically for the treatment of numerous diseases and injuries through regenerative medicine approaches. AF-SCs exhibit lower immunogenicity, which reduces the risk of immune rejection after transplantation. This makes them particularly suitable for clinical applications, where immunological compatibility is a significant concern. The ability of these cells to differentiate into different types of neuronal cells, to support tissue regeneration and to modulate the inflammatory properties of the CNS opens up new avenues for innovative treatments in regenerative medicine and neurological therapies.

## Figures and Tables

**Table 1:** Sequences of the primers used in rt-PCR.

Gene	Gene Name	Primer Sequence
ACTB Forward	Actin Beta	GTGCGTGACATTAAGGAGAA
ACTB Reverse	Actin Beta	ATGGAGTTGAAGGTAGTTTCGT
KCNMA1 Forward	Potassium calcium-activated channel subfamily M alpha 1	CTAATTCCCAAGGGTTCACAC
KCNMA1 Reverse	Potassium calcium-activated channel subfamily M alpha 1	GCTTTGCAGAACAGATCACCA

## Data Availability

The original contributions presented in the study are included in the article, further inquiries can be directed to the corresponding authors.
